# Targeting the epidermal growth factor receptor using IgM antibodies: toward next generation cancer immunotherapy

**DOI:** 10.3389/fimmu.2025.1733907

**Published:** 2026-01-09

**Authors:** Kamolrat Somboon, Peter J. Bond, Firdaus Samsudin

**Affiliations:** 1 Bioinformatics Institute (BII), Agency for Science, Technology and Research (ASTAR), Singapore, Singapore; 2 Department of Biological Sciences, National University of Singapore, Singapore, Singapore

**Keywords:** cancer, Cetuximab, EGFR, IgM, Matuzumab, monoclonal antibody

## Abstract

Immunoglobulin G (IgG) monoclonal antibodies dominate current cancer immunotherapy but face challenges including resistance development, limited tumor penetration, and suboptimal avidity. In contrast, the pentameric or hexameric architecture of immunoglobulin M (IgM) offers up to twelve antigen-binding sites and potent complement activation, positioning IgM as a promising next-generation therapeutic scaffold. Here, we present integrative structural modeling and multiscale molecular dynamics simulations of IgM versions of Cetuximab and Matuzumab targeting the epidermal growth factor receptor (EGFR), a clinically validated oncogenic driver. Our analyses reveal that IgM antibodies maintain a rigid, glycan-stabilized Fc core while their Fab domains exhibit high mobility, enabling multivalent EGFR binding. Compared with IgG, IgM antibodies demonstrated enhanced binding avidity, prolonged receptor engagement, and slower dissociation kinetics. These properties suggest superior therapeutic durability and potential to overcome current limitations of IgG-based therapies. By providing mechanistic insight into how IgM isotypes can improve therapeutic engagement with tumor-associated antigens, our study supports the development of IgM antibodies as a new class of cancer immunotherapies.

## Introduction

Monoclonal antibodies (mAbs) have revolutionized cancer therapy by selectively targeting antigens on cancer cells. This specificity allows mAbs to deliver cytotoxic agents, mark cells for immune destruction, or inhibit growth signals, thereby limiting tumor progression while minimizing damage to healthy tissues (1). Unlike traditional therapies like chemotherapy and radiation, which can cause significant collateral damage, mAbs offer improved efficacy and fewer side effects (2). Furthermore, mAbs can be engineered to enhance their immune-mediated effects or to carry therapeutic agents directly to cancer cells (3). While immunoglobulin (Ig) isotype G (IgG) antibodies are widely used in cancer therapy for their longevity and ability to activate immune responses (4), Ig isotype M (IgM) antibodies are gaining attention. The pentameric or hexameric architecture of IgM in principle allows for higher avidity due to multivalent antigen binding and an enhanced ability to activate the complement system, making it highly promising for cancer immunotherapy (5) (6) (7),,. Additionally, IgG therapies face challenges such as resistance development, limited tumor penetration, and severe immune-related adverse effects (8) (9) (10) underscoring the need for novel alternatives like IgM, which could potentially overcome these obstacles due to its distinct properties.

IgM represents the first-line antibody isotype expressed during an immune reaction, serving an important role in the primary immune response due to its strong antimicrobial properties and role in activating the complement system (11) (12),. Structurally, IgM antibodies can be configured into either pentamers or hexamers, which are complexes of five or six subunits, respectively. These subunits are linked by disulfide bridges, creating a stable, multimeric complex (13) (14),. Each IgM subunit comprises four polypeptide chains: two heavy chains and two light chains. The heavy chains contain four constant domains (Cμ1, Cμ2, Cμ3, Cμ4) and one variable domain (VH) for antigen binding. The light chains comprise one constant domain (CL) and a variable domain (VL). Each variable domain (VH and VL) contains three complementarity-determining regions (CDR1, CDR2, and CDR3), which are hypervariable loops responsible for the exquisite specificity and affinity of the antibody for its target antigen (15). Importantly, the Cμ2 domain in IgM replaces the hinge region found in other antibody isotypes such as IgG, providing rotational flexibility to the fragment antigen-binding (Fab) domains of these heavy chains (16). This arrangement permits the antigen-binding sites to adjust flexibly, crucial for the stable and simultaneous binding of multiple antigens. IgM is typically organized in a pentameric form, connected by a joining (J)-chain. This essential short polypeptide not only stabilizes the structure but also facilitates the polymerization of IgM molecules, significantly enhancing its functional role in immune responses (17) (18).,. Although their multimeric structure allows binding to multiple antigens simultaneously, enhancing their effectiveness over IgG antibodies, their complex structure presents challenges to researchers in rational engineering due to the lack of available high-resolution structural data.

One of the most attractive targets for cancer therapy is the epidermal growth factor receptor (EGFR), which plays a crucial role in cell proliferation and survival and is often overexpressed in numerous cancers (19) (20) (21),,. Cetuximab and Matuzumab, which target EGFR, have shown significant efficacy in treating cancers such as colorectal cancer as well as head and neck squamous cell carcinoma (22) (23),. Developing IgM versions of these antibodies could enhance their therapeutic effects due to IgM’s superior avidity and complement activation properties. Thus, the primary goal of this research is to develop and validate an accurate structural model of IgM monoclonal antibodies for Cetuximab and Matuzumab to explore their unique dynamics and receptor-binding capabilities compared to their IgG counterparts.

In our previous work, we built a full-length IgM model that demonstrated the potential of IgM antibodies in targeting cancer antigens, specifically on the ability of Pertuzumab IgM to bind multiple human epidermal growth factor receptor 2 (HER2) simultaneously, thus enhancing its inhibitory effect compared to IgG (24). In the current study, we present a more comprehensive computational analysis of IgM monoclonal antibodies, particularly on Cetuximab and Matuzumab targeting the EGFR. We refined our full-length IgM model to include the recently published cryo-electron microscopy (cryo-EM) structure of the human IgM fragment crystallization (Fc) region in complex with the J-chain (25) as well as N-glycans from glycomics studies (26) (27) (28) (29), (30), thus enhancing biological relevance and functional accuracy. We employed coarse-grained (CG) molecular dynamics (MD) simulations and enhanced sampling methods to explore their dynamic behavior and binding interactions under physiological conditions. Our simulations revealed that Cetuximab and Matuzumab IgM antibodies exhibit significantly enhanced binding avidity and stability compared to their IgG counterparts. Additionally, the refined IgM model demonstrated a more flexible Fab region, which is crucial for effective multivalent binding and improved therapeutic efficacy. Through this approach, our research contributes significantly to antibody-based cancer therapies, challenging existing treatment models and paving the way for innovative next-generation antibody therapeutics.

## Results

### Conformational stability and dynamics of integrative IgM models

**Figure 1:**
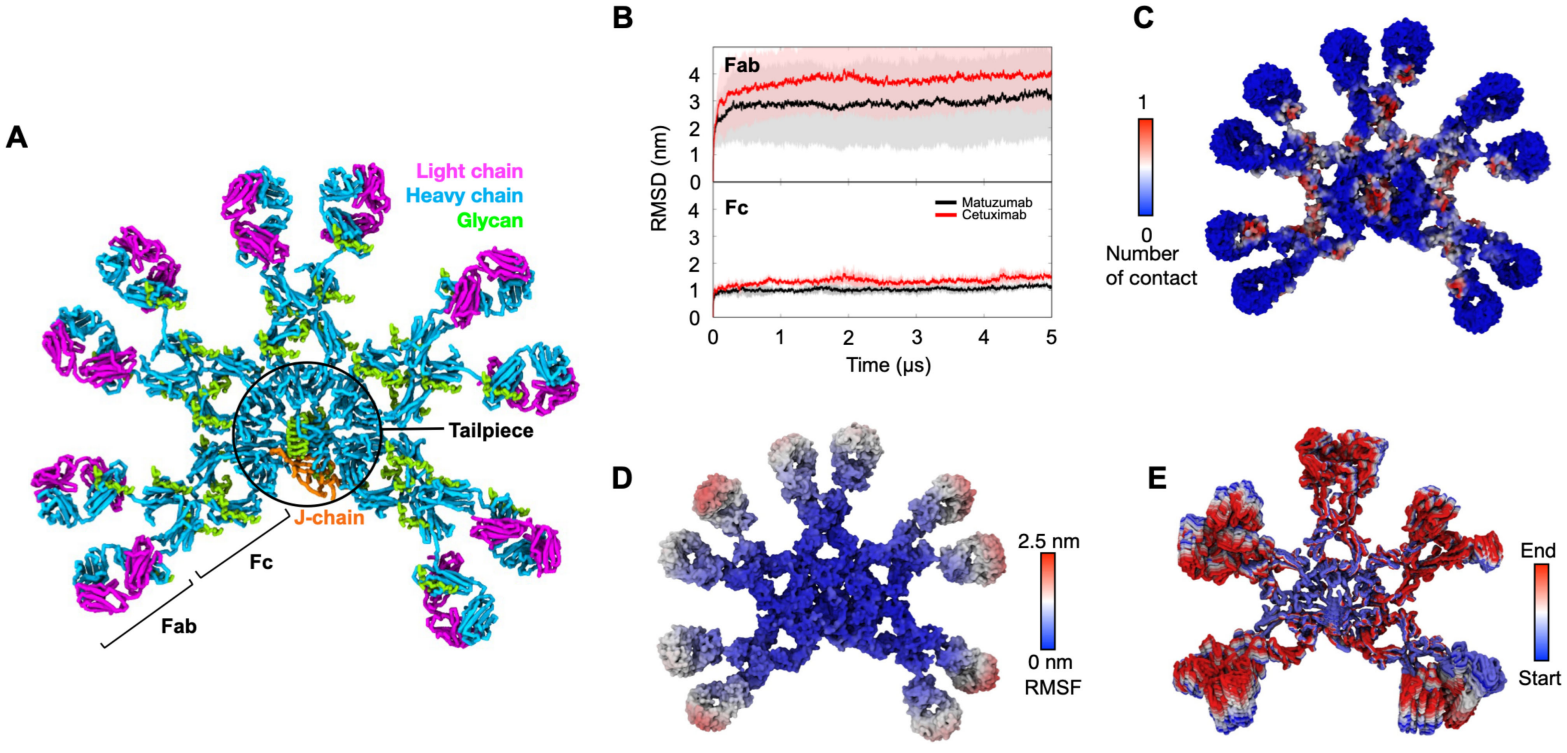
Completing the IgM model contributes to enhancing the stability of the overall IgM structure. **(A)** CG model of glycosylated Cetuximab IgM. Heavy chain in cyan; light chain in magenta; J-chain in orange; glycans in green. **(B)** The average root mean square deviation (RMSD) of the backbone particles for Fc and Fab of Matuzumab (in black) and Cetuximab (in red) IgM is represented. The solid lines depict the mean values obtained from three independent 5 µs simulations, while the shaded regions denote the standard deviations. **(C)** The average number of contacts made by each residue of the IgM with glycans from the simulations. The distance cut-off used for contact analysis is 0.6 nm. **(D)** Root mean square fluctuation (RMSF) values for individual residues of Cetuximab IgM from a single 5 µs simulation depicted on the protein surface. **(E)** The first principal motion of all Cetuximab backbone atoms from one of the 3 independent simulations was identified through principal component analysis (PCA), accounting for 37.26% of the total structural variance.

We first built IgM models of Cetuximab and Matuzumab by integrating data from the cryo-EM structure of human IgM Fc pentamer in complex with the J-chain and the crystal structures of the Fab domains, as well as glycomics data for the antibodies (details in Materials and Methods) (**Figure 1A**). We subsequently ran three replicates of 5 µs coarse-grained (CG) MD simulations to assess the stability of the models. Initially, we evaluated the conformational stability of individual Ig domains within the IgM models by calculating the average backbone root mean square deviation (RMSD) of the Fab and Fc domains separately, relative to their starting structures. Both domains exhibited low RMSD and rapidly converged (~0.8 nm for the Fc domain and ~0.2 nm for the Fab domain, as shown in **Supplementary Figure 1**), indicating conformational stability with no intradomain structural changes, as expected due to the elastic network models (ENMs) imposed to maintain the protein folds. This is in agreement with our previous CG simulations of IgM protomers, where individual Ig domains remained stable due to disulphide bonds and the implementation of ENMs (24).

To explore the conformational dynamics of the entire IgM structure, the average backbone RMSD values were next calculated for the Fab and Fc domains, but after least-squares fitting to the complete IgM structure. Overall, the RMSD values of both domains rapidly converged within the first microsecond. Higher RMSD values and fluctuations were observed in the Fab domains (RMSD range ~2–4 nm), while the RMSD values of the Fc domain remained at ~1 nm (**Figure 1B**). The significantly higher RMSD values on the Fabs domains indicate that they are more mobile with respect to the Fc domain. On the other hand, the Fc domain exhibited greater rigidity, attributed to the presence of glycans on the Cµ2, Cµ3, and Cµ4 domains, which interacted predominantly with the Fc and tailpiece regions (**Figure 1C**). This interaction further reduced dynamics within the Fc domain, as confirmed by calculation of the root mean square fluctuation (RMSF) patterns across the protein (**Figure 1D**). Additionally, the presence of disulphide bridges linking each subunit contributes to the rigidity of the Fc region. These findings are in agreement with Principal Component Analysis (PCA) conducted to detect significant, dominant motions within IgM by characterizing the motion along the first eigenvector for the protein backbone. PCA highlighted the higher flexibility of the Fab domain compared to the Fc domain (**Figure 1E**; **Supplementary Figure 2**). Overall, the conformational dynamics observed in these IgM antibodies are consistent with our previous simulations of Pertuzumab and Trastuzumab, which demonstrated flexibility within the Fab region while maintaining rigidity in the Fc region (24).

### Accessibility to the CDRs on IgM

**Figure 2:**
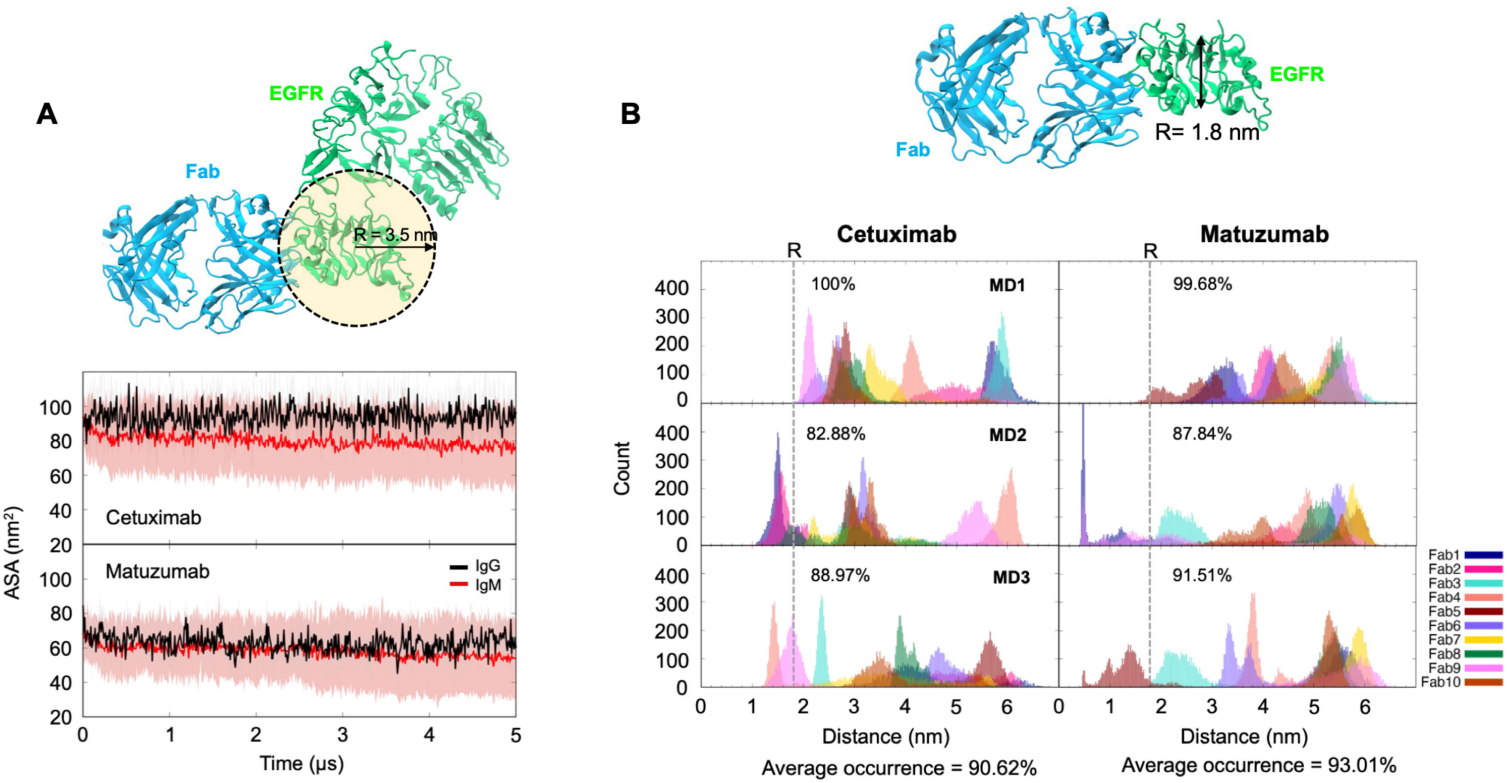
The likelihood of simultaneous binding of EGFR to Cetuximab and Matuzumab IgM antibodies. **(A)**. *Top*: The methodology used to estimate the probe size for CDR Accessible Surface Area (ASA) calculations in the EGFR-Matuzumab complex. The cut-off radius was set based on half of the maximum width (3.5 nm) of the EGFR binding domain. The crystal structure of the extracellular domain of EGFR from PDB:4UV7 was superimposed on to the EGFR-Matuzumab complex (PDB: 3C09) to facilitate these calculations. *Bottom:* The comparison of CDR ASA values between the IgM (red) and its IgG (black) counterparts. Average values calculated from all the analyzed Fabs are depicted with thick lines, while standard deviations are represented by shaded areas. **(B)**. *Top:* The crystal structure of the EGFR-Matuzumab complex (PDB: 3C09). The cut-off radius for analysis was determined from the minimal width (1.8 nm) of the EGFR binding domain. This radius was subsequently used to calculate the distance distribution. *Bottom:* The distance distribution between the center of mass of the CDR on the Fab domain and the nearest residue within the antibody structure. The data represents different colors for each of the ten Fab domains. These results were derived from three independent simulations of each IgM antibody. The dotted line marks the critical distance threshold required for EGFR binding.

Interestingly, we observed that the IgM Fabs experienced decreased accessibility to the CDRs due to the crowdedness of the Fab domains and their inherent flexibility. PCA calculations unveiled significant mobility within the Fab domains, leading to self-association with adjacent Fabs *via* non-specific, long-lived interactions (**Figure 1E**). This inter-domain interaction effectively limited access to the critical CDR, which is essential for antigen recognition (**Supplementary Figure 3**). To further elucidate the dynamics of the Fab domains and their impact on CDR accessibility, we computed the accessible surface areas (ASAs) of the CDR within each Fab domain from the unbound simulations. The selection of a 3.5 nm probe size was guided by the estimated dimensions of EGFR’s domain III, which binds to the antibody (**Figure 2A**). We acknowledge that using a spherical probe will not fully capture the anisotropy, inter-domain flexibility, or conformational motions of the EGFR ECD. However, the probe will allow for an approximate measurement of the Fab accessibility to compare between the IgM and IgG isotypes. Notably, we found that the average ASAs of Cetuximab and Matuzumab IgM Fab domains were slightly lower than those of equivalent IgG isotypes (~80 nm^2^ and 60 nm^2^, respectively) (**Figure 2A**). However, the differences in ASA values between IgM and IgG are small and within the error bars due to the dynamic nature of the Fab domain; hence, the ASA differences caused by Fab-Fab interactions are likely insignificant.

To exploit the large number of Fab domains in IgM, the Fab domain must be able to accommodate simultaneous binding of multiple receptors. To measure the likelihood of simultaneous receptor binding, we measured the spatial requirements for multiple binding based on the size of the EGFR ectodomain (ECD). We calculated the distance distribution between the center of mass (COM) of the CDR and the nearest Fab or Fc domains sampled during our unbound simulations. We established that a minimum distance of approximately 1.8 nm is required for EGFR binding (**Figure 2B**). Our unbound simulations spanned 5 µs, with snapshots recorded every 1 ns, resulting in a total of 5,000 frames. Given that each IgM comprises ten Fabs, the cumulative configurations from three independent runs totalled 150,000 Fab configurations. The resulting distribution of distances is shown in **Figure 2B**, providing valuable insights into the accessibility of the CDR region for potential simultaneous EGFR binding. We found that the average probabilities of EGFR simultaneous binding to the CDR of Cetuximab and Matuzumab IgM from our simulations were 90.62% and 93.01%, respectively. Despite the observed decrease in CDR accessibility compared to the IgG counterparts due to Fab-Fab interactions, the IgM Fab domains still exhibited a high capacity to accommodate EGFR interaction due to the dynamic nature of the Fab domains. For comparison, our previous study showed that less than 5% of the conformations sampled by Trastuzumab IgM in our CG simulations would allow for simultaneous HER2 binding (24). Collectively, both Cetuximab and Matuzumab IgMs are still likely able to accommodate simultaneous binding and have improved avidity compared to the equivalent IgGs.

### Simultaneous binding of EGFR to IgM

**Figure 3:**
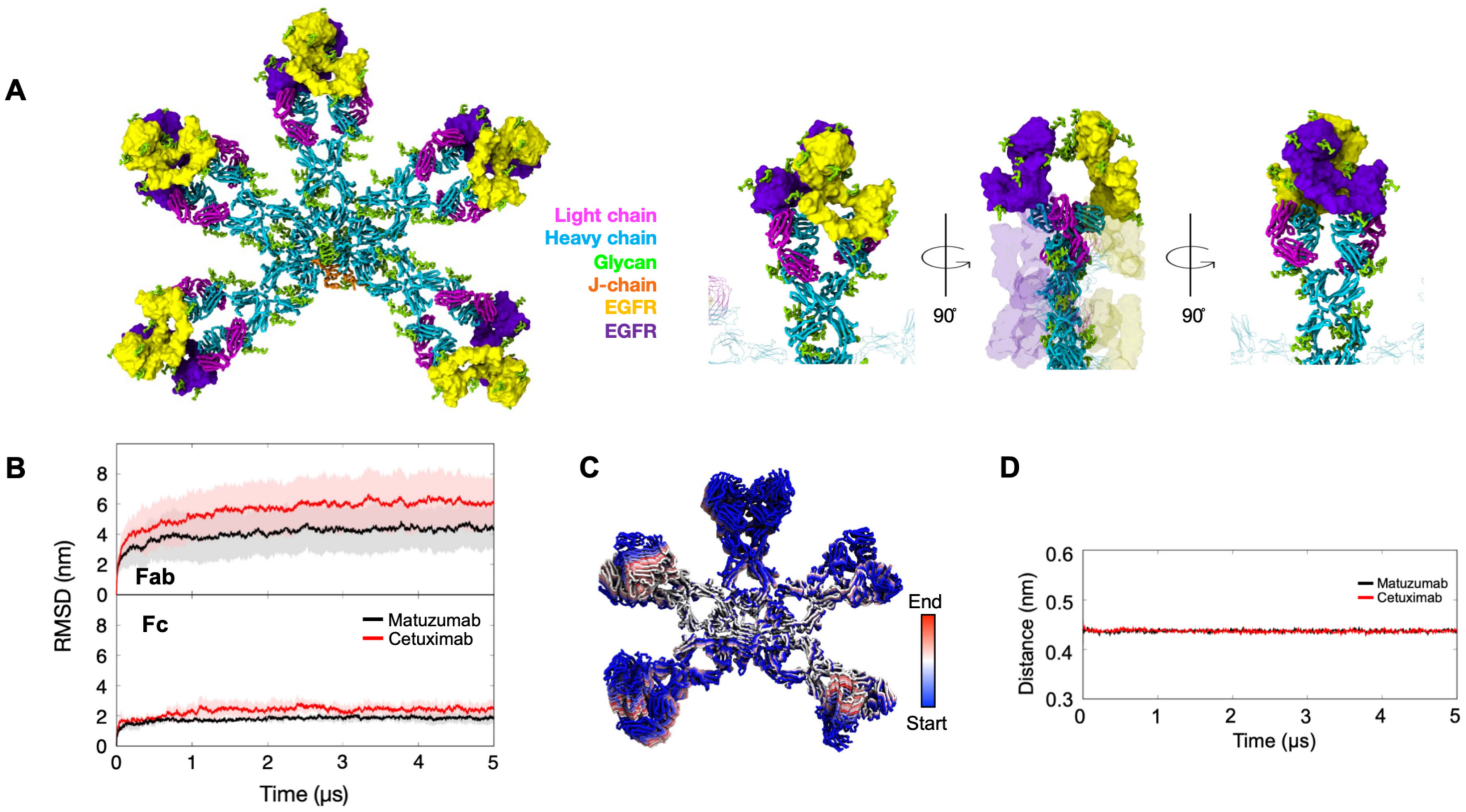
The binding of EGFRs to the Cetuximab and Matuzumab IgM. **(A)***Left*: The alignment of ten EGFRs (colored in yellow and purple) onto the Matuzumab IgM model. The alignment is based on the monomeric binding site, as described in a previous study (22) (23),. Heavy chains, light chains, J-chains, and glycans are colored cyan, magenta, orange, and green, respectively. Ten EGFRs (colored in yellow and purple) were positioned onto the Matuzumab IgM model based on the monomeric binding site, as reported previously. *Right:* Close-up images of a single subunit of Matuzumab IgM with EGFRs bound. For clarity, the image displays only two EGFR units and one IgM. The IgM unit is shown in licorice representation, with the heavy and light chains colored cyan and magenta, respectively. The EGFRs are depicted in surface representation, colored yellow and purple. **(B)** The average RMSD of the backbone particles, aligned to the overall IgM backbone, for the Fc and Fab regions of Matuzumab (in black) and Cetuximab (in red) antibodies. The solid lines illustrate the mean RMSD values derived from three independent simulations, each 5 microseconds in length. The shaded areas around these lines indicate the standard deviations, showcasing the variability of the RMSD across the simulations. **(C)** The first motion of all Matuzumab backbone atoms from one of the 3 independent *bound* simulations, as determined through PCA. The progression of the motion is represented by a color gradient, starting from blue and transitioning through white to red. **(D)** The average of the minimum distance between the centers of mass of the CDR region on each Fab and the bound EGFR. The data were obtained from three independent bound simulations of Cetuximab IgM (red) and Matuzumab IgM (black).

We next evaluated the avidity of these IgM models by modeling the binding of Cetuximab and Matuzumab IgM to the EGFR ECD. Structural alignment of each of the ten Fab domains in the IgM pentamer with the crystal structures of the Fab domains bound to EGFR was performed (details in Materials and Methods section). Despite Cetuximab and Matuzumab IgM binding to different epitopes on EGFR ECD, our structural alignment indicated that simultaneous binding of all ten Fab domains of the IgM pentamer to multiple receptors is indeed feasible for both antibodies (**Figure 3A**; **Supplementary Figure 4**). This finding is in contrast with our previous study on Trastuzumab IgM, where simultaneous binding of HER2 to multiple Fabs was not possible due to steric clashes between the ECD of bound HER2 receptors with neighboring Fab domains (24).

To evaluate the stability of simultaneous receptor binding, we conducted CG MD simulations of the Cetuximab and Matuzumab IgM pentameric models, with all ten Fab domains bound to the EGFR ECD. Our analysis revealed stable binding between the two IgMs and EGFR throughout the simulations. Similar to observations for the IgM unbound systems, the intradomain RMSDs stabilized after approximately 150 ns, indicating the stability of individual Ig domains (**Supplementary Figure 1**). However, when the simulated structures were least-square fitted to the overall backbone of the initial IgM model, we observed increased RMSD values and fluctuations in the Fab domains (range of ~4–6 nm) and the Fc domains (range of ~2–3 nm), suggesting heightened mobility compared to the unbound simulations (**Figure 3B**). Intriguingly, despite the large distance between the receptor binding epitope and the Fc region, we still observed an increase in the RMSD of the Fc domain in the receptor-bound state compared to the unbound state, suggesting potential allosteric effects caused by receptor binding. Similar to the unbound simulations, our PCA demonstrated significant mobility in the bound Fab domains (**Figure 3C**). Remarkably, even with the dynamic nature of the Fab domains, all antigens remained bound to IgM throughout the simulations, as demonstrated by the stable and low distance measured between the centers of mass of EGFR and CDR of IgM throughout the 5 µs simulations (**Figure 3D**). These results highlight the robustness of simultaneous receptor binding to the IgM antibodies.

### Binding affinity of EGFR to IgM

**Figure 4:**
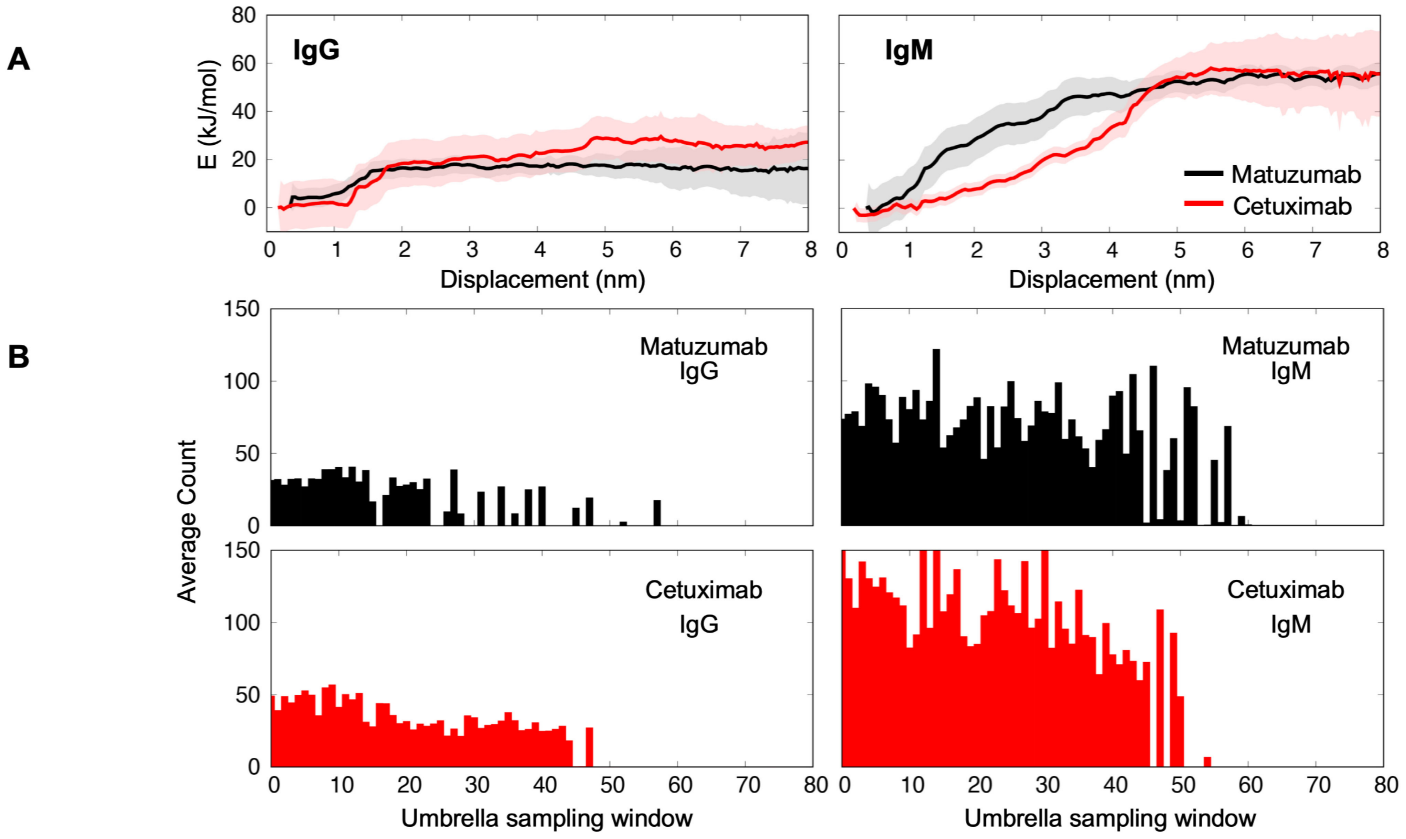
PMF calculation of EGFR binding to IgMs and IgGs. **(A)** The comparison of PMF for IgG (left) and IgM (right) antibodies. The data for Matuzumab IgM and its counterpart are represented in black, while the results for Cetuximab IgM and its counterpart are shown in red. **(B)** The number of molecular contacts calculated between EGFR and IgG (left) or IgM (right) antibodies across each US window. The results for Matuzumab are shown in black, while those for Cetuximab are displayed in red.

We hypothesized that the large number of Fab domains in IgM enhances the probability of interaction with the EGFR ECD, and hence should increase the overall effective binding affinity. To accurately quantify the binding energies, we conducted potential of mean force (PMF) calculations of EGFR binding to the IgM Cetuximab and Matuzumab models within an umbrella sampling (US) framework. The bound EGFR ECD was pulled away from the Fab domain using steered MD simulations (**Supplementary Figure 5**). Subsequently, US simulations were conducted along the dissociation pathway. We then compared the PMF profiles to that from similar US simulations using the equivalent IgGs. We observed higher binding affinities (~60 kJ/mol) for Cetuximab and Matuzimab IgMs compared to their IgG counterparts (~20–30 kJ/mol) (**Figure 4A**). Interestingly, the convergence for PMF calculations using IgMs occurred at a distance of approximately 5 nm away from the IgM, notably further than that using the IgGs (~1.5 nm), suggesting more prolonged interactions between EGFR and the IgMs.

Further analysis based on averaging the number of interactions between the antibody and the EGFR in each US window revealed that both IgM variants engaged in a higher number of interactions with the EGFR (**Figure 4B**), suggesting a slower dissociation rate (K_off_) of IgM binding to the EGFR in solution compared to IgG. These findings highlight the unique binding characteristics of Matuzumab and Cetuximab in their IgM isotype attributed to the formation of more favorable contacts with the EGFR. Collectively, these simulations agree with our hypothesis that the higher number of Fabs in the IgM could increase binding avidity to EGFR, and therefore be a more effective therapeutic option compared to the IgG isotype.

## Discussion

Our comprehensive study introduced newly developed models of full-length IgM antibodies, specifically for Cetuximab and Matuzumab, providing crucial insights into their conformational dynamics and receptor-binding abilities. The findings revealed that the IgM isotypes of these antibodies exhibit superior binding affinity and stability compared to their IgG counterparts, highlighting their promising therapeutic potential in cancer treatment.

Our refined IgM models, combining critical structural elements, including elastic network models, N-glycans, as well as the tailpiece and the J-chain structures, enhance the overall structural stability while preserving the essential flexibility of the Fab regions. The use of an elastic network in our CG model ensures that, despite the reduced resolution of the model due to the substantial size and complexity of the IgM, the antibody maintains its functional conformation under physiological conditions. The overall structure aligns well with cryo-microscopy data (31) that depict a planar Fc domain. However, incorporating the elastic network may restrict significant conformational changes in the antibody, as we observed no pronounced bending in the Fc region. This finding contrasts with a previous study indicating distortions in the Fc region, resulting in a dome-like shape (32).

Besides the rigidity introduced by the elastic network, the presence of glycans at the five putative N-linked glycosylation sites on the heavy chains and one on the tailpiece region significantly stabilizes the dynamics of the Fc domain, mainly affecting the Cµ3, Cµ4, and the tailpiece regions (30). This is due to extensive glycan interactions with these areas of the Fc domain. Prior wet-lab experiments and computational studies support the idea that glycosylation reduces protein dynamics without causing significant structural changes (33) (34) (35),,. Glycosylation also plays a critical role in modulating antibody effector functions such as antibody-dependent cellular cytotoxicity and complement activation (36) (37),. However, these glycans might hinder the ability of Cetuximab and Matuzumab to bind multiple EGFRs due to prolonged interactions between the glycan positioned at Cµ1, which is located between the Fab domains of adjacent IgM subunits, and other glycans. Although this glycan did not form any interactions with the CDR regions in our simulations due to its considerable distance from the antigen-binding site, it could still restrict the flexibility of the Fab domain, thereby affecting the binding efficiency of IgM. Hence, while individual Fab domain retains local conformational mobility, their global motions are constrained by the IgM architecture, including the presence of nearby N-glycans.

The inclusion of the J-chain and tailpiece in our IgM models significantly refine their structural and functional characteristics. The J-chain links multiple monomers together, thereby enhancing structural integrity (38). Simultaneously, the tailpiece expands the functional scope of the Fc domain, providing a scaffold for complement activation and interaction with Fc receptors (38) (39),. More than just a structural extension, the tailpiece actively increases the rigidity of the Fc domain. This modification allows the Fab regions to move more independently and agilely, potentially increasing the antibody’s binding affinity and versatility, thereby enhancing its therapeutic efficacy.

Allosteric regulation plays a crucial role in antibody function by modulating binding affinity and specificity, which are essential for effective immune responses (40) (41) (42) (43). While previous studies have explored allosteric interactions within IgG and IgA, demonstrating potential communication between Fab and Fc domains upon antigen binding (40) (41) (44) such mechanisms in multimeric IgM remain less understood. Our research extends these findings by examining how conformational changes propagate through IgM structures upon interaction with antigens. Specifically, we observed an increase in the RMSD values in both Fab and Fc domains when bound to EGFRs compared to unbound simulations, suggesting long-range dynamic structural adjustments. Interestingly, the individual Fab domains exhibited independent motion, not influencing the movement of adjacent domains, corroborating findings from our previous multiscale simulation study (24). Contrasting with earlier observations (24), the increased RMSD values in the Fab and Fc domains, compared to their unbound states, hint at potential allosteric interactions mediated through the Fc region. However, the presence of an elastic network and glycans may restrict these movements, as previously discussed, potentially limiting the extent of conformational changes necessary for effective allostery. This study highlights the complex nature of allosteric regulation in IgM and underscores the need for further investigations to fully understand the mechanistic basis of its regulatory roles.

IgM monoclonal antibodies (mAbs) were among the earliest antibody therapies tested in clinical trials (45). Recent research shows that they are effective across various animal models, including those involving primates (46) (47),. This research highlights the increasing exploration of IgM for its distinct qualities. Despite promising results in animal studies, IgM antibodies have not progressed much to human trials for cancer treatment (48) (49) (50) (51). This delay is mainly due to several obstacles. Typically, these antibodies are derived from natural sources and consist of germ-line gene sequences that lack significant somatic mutations, leading to reduced affinity and specificity (52). Moreover, the substantial size of IgM molecules may impair their ability to penetrate tissues effectively, a disadvantage compared to smaller antibodies like IgG or IgE. For IgM-based therapies to move from laboratory settings to clinical practice, significant advancements in manufacturing processes, thorough testing, and obtaining of regulatory approvals are necessary. These steps are costly and time-consuming but essential for successfully adopting IgM therapies in clinical settings.

While our simulations demonstrate the strong avidity and sustained receptor engagement of IgM, it is also important to acknowledge a recognized limitation of IgM-based therapeutics: their large molecular size. Pentameric IgM (~970 kDa) diffuses more slowly through the dense extracellular matrix of solid tumors than IgG, potentially restricting deep tissue penetration. Thus, the same multivalency that enhances binding may also constrain tumor accessibility. Engineering strategies, such as reduced-valency or activation-dependent IgM formats, may help overcome these limitations while preserving multivalent binding advantages.

The large size of IgM also limits the types of computational studies that are feasible. An atomic-resolution simulation of IgM bound to full-length EGFR ECD would contain tens of millions of atoms, which is prohibitively expensive to perform. Thus, in this study, the coarse-graining approach, which reduced the system size to ~500k provides a practical framework for comparing the IgM to the IgG isotype. We note that this approach resulted in the loss of atomic-level interaction details such as hydrogen bonds or pi-stacking interactions; however, the Martini CG forcefield has been extensively used to study protein-protein interactions, especially for larger systems (53) (54),. In our PMF calculations, we employed only domain III of the EGFR ECD, which contains the epitopes for Cetuximab and Matuzumab, to aid convergence. As such, contributions of other domains would not be captured by our PMF calculation. However, due to the inter-domain flexibility and the much larger size, longer simulations would have been required for adequate conformational sampling to reach convergence in PMF calculations if the full-length EGFR ECD was used.

As we explore the future of cancer therapy with IgM antibodies, our recent simulation results further emphasize their potential. Our results demonstrate that IgM can simultaneously bind to multiple antigens on the surface of cancer cells, a capability not typically observed with traditional IgG antibodies. This multivalent binding could provide a significant advantage, potentially leading to more robust and effective targeting of cancer cells (55). The ability of IgM to engage multiple antigens simultaneously would enhance its therapeutic efficacy and offer a stronger blockade against cancer cell growth and proliferation. In the context of cetuximab specifically, recent clinical evidence supports the biological relevance of EGFR-directed antibody mechanisms, as cetuximab has been shown to rapidly deplete tEGFR-engineered immune cells *in vivo*, demonstrating potent EGFR-dependent activity (54). Our findings are encouraging as they suggest that IgM antibodies could outperform conventional IgG in certain therapeutic scenarios, particularly in targeting cancers with high antigen variability or density. This property of IgM could pave the way for developing more potent and specific cancer treatments, marking a significant step forward in the evolution of antibody-based therapies.

## Conclusions

In conclusion, our computational study has successfully led to development and refinement of a model for IgM antibodies, particularly for Cetuximab and Matuzumab, providing valuable insights into their conformational dynamics and enhanced receptor-binding capabilities. In future, these findings will be calibrated and tested in physiologically relevant model studies. Thus, it is crucial to note the limitations of our current approach, particularly the absence of modeling of IgM binding with full-length EGFR within a realistic membrane environment. For example, factors such as receptor density on cancer cell membranes, along with cellular characteristics like membrane morphology and undulations could influence the potential for multiple binding. Addressing these aspects, alongside expanding the scope of our simulations to capture more biologically accurate contexts, represents a key priority for future studies. This underscores the importance of refining our technique and expanding the scope of our simulations in subsequent research. Moreover, the application of our computational simulations in practical therapeutic contexts still relies on *in vivo* experimentation and clinical trials. These essential next steps will not only confirm the effectiveness and safety of our modeled IgM antibodies but also facilitate their potential clinical implementation. By shedding light on the conformational dynamics and functional capacities of IgM antibodies, our research makes a significant contribution to ongoing research in antibody-based therapies, opening chances for treating various diseases, particularly cancer.

## Materials and methods

### Integrative modeling of IgM antibodies in an unbound state

The full-length IgM models for Cetuximab and Matuzumab were built using structural data available in the PDB: i) the X-ray crystal structures of Cetuximab and Matuzumab Fab bound to the human EGFR extracellular domains (ECDs) (PDB: 1YY9 (56) and 3C09 (22), respectively), ii) the cryogenic electron microscopy (cryo-EM) structure of the human IgM Fc pentamer (PDB: 6KXS) (25), and iii) the crystal structure of mouse Cµ2 domain (PDB: 4JVU) (57). A total of 10 full-length IgM pentamer models in complex with the J-chain were constructed for each antibody. The best models were selected based on having the fewest Ramachandran outliers.

### Integrative modeling of IgG antibodies in an unbound state

Cetuximab and Matuzumab IgG models were constructed based on the X-ray crystal structure of human IgG1-Fc (PDB:5JII) (58) and their respective Fab domains described above. The alignment of these domains onto the previously reported crystal structure of a complete IgG2 antibody (PDB:1IGT) (59) was performed using PyMOL (60). Subsequently, Modeller (61) was utilized to introduce the missing residues and linkers between the domains. Ten models for both Cetuximab and Matuzumab IgGs were generated, with the models exhibiting the fewest Ramachandran outliers chosen for simulations.

### Integrative modeling of IgM and IgG antibodies in a bound state

To model the bound systems of IgM and IgG antibodies in complex with the EGFR, we obtained the complete EGFR ECD structure from a cryo-EM structure (PDB:7SYD) (62). The ECDs of EGFR were aligned with our pre-selected models of IgM and IgG, as mentioned above. This alignment was performed using PyMOL based on the crystal structures of the Fab segments of the two antibodies bound to the human EGFR ECDs described above.

### Equilibrium coarse-grained molecular dynamics simulations

We conducted coarse-grained (CG) molecular dynamics (MD) simulations on the chosen antibody models using GROMACS 2018 (www.gromacs.org) (63). CG models were generated using the Martini 2.2 force field enhanced by the ElNeDyn elastic network (64) (65), to maintain the secondary and tertiary structure. We then introduced glycan molecules using the extended Martini 2.2 parameter for N-glycans (66). Based on glycomics data, for the IgG models, Cetuximab and Matuzumab heavy chains each received two core-fucosylated complex glycans: one at the Fab domain and the other at the Fc domain (26) (27) (28) (29). The IgM models were modified to include four glycans per monomer: one core-fucosylated complex glycan at Cμ2, one core-fucosylated complex glycan and one high-mannose oligosaccharide at Cμ3, and one high-mannose oligosaccharide at the tailpiece, with an additional a high-mannose oligosaccharide on the J-chain (67). Following glycosylation, we solvated the proteins with the standard Martini water model and 0.15 M Na+ and Cl- ions to mimic physiological conditions. To resolve any steric clashes, we performed energy minimization for 5,000 steps using the steepest descent method. A 100 ns equilibration simulation was then performed with protein backbone atoms restrained using a force constant of 1,000 kJ mol^-1^ nm^-2^. We maintained a temperature of 310 K with a velocity-rescaling thermostat (68) and a pressure of 1 atm with a Berendsen barostat (69), each set to time constants of 1 ps and 5 ps, respectively. For the production runs, three 5 µs independent simulations were performed for each antibody model, varying initial velocities. The pressure of the system was maintained by a Parrinello-Rahman barostat (12 ps time constant) (70), and the temperature was controlled using a similar thermostat as the equilibration phase. We truncated non-bonded and short-range electrostatic interactions at 1.1 nm using a potential shift Verlet cut-off, while long-range electrostatics were handled by the reaction field method. A summary of all production runs is shown in **Supplementary Table 1**. Analysis was performed using the GROMACS package. Molecular visualization was created with PyMOL (60) and Visual molecular dynamics (VMD) software (71).

### PMF calculation of EGFR binding to IgM and IgG

US simulations were utilized to examine the potential energy across the binding pathway between the antibodies and the EGFR. We focused these simulations along the z-axis, which is the principal axis of the IgM model, and limited our sampling to domain III of EGFR to help reach convergence within the timescale of our simulations. For initial setup, we positioned this EGFR domain III on the fifth Fab of both Cetuximab and Matuzumab IgMs (**Supplementary Figure 5**), using structural alignment to PDB 1YY9 (56) and 3C09 (22), respectively. Each system was solvated and added ions followed by a 100 ns equilibration simulation using the same parameters described above. We then performed constant velocity pulling simulations along the y-axis to generate initial inputs for US MD simulations. A harmonic spring with a force constant of 1,000 kJ mol^-1^ nm^-2^ was applied to the center of mass of the EGFR. This setup was pulled at a rate of 0.5 nm/ns for 100 ns. Snapshots along the y-directions were then selected as US windows with an equal separation of 0.1 nm between windows (from 0 to 8 nm). Each US window underwent a 500 ns MD simulation, applying a harmonic bias potential with a 1,000 kJ mol^-1^ nm^-2^ force constant on the center of mass of the substrates along the y-axis, without any restraints in the x- and z-planes. To assess adequate sampling, we evaluated the overlap of histogram profiles visually, as depicted in **Supplementary Figure 6**. We calculated the potential of mean force (PMF) profiles using the Weighted Histogram Analysis Method (WHAM) (72) as implemented in GROMACS. Additionally, we estimated the correlation time for each simulation window and calculated the Bayesian bootstrapping error of each PMF using the built-in GROMACS tools (73). The PMF profiles are presented in **Figure 4**. For PMF calculations of EGFR binding to the IgGs, we similarly placed domain III of EGFR at the initial position on the first Fab domain (**Supplementary Figure 5**). The same parameters as in the IgM simulations were applied, except for the harmonic bias potential that was applied along the x-axis instead of the y-axis.

## Data Availability

The datasets presented in this study can be found in online repositories. The names of the repository/repositories and accession number(s) can be found here: https://zenodo.org/records/17188634.

## Supplementary material

Supplementary Table 1 List of all production simulations

Supplementary Figure 1 RMSD Profiles for Matuzumab and Cetuximab IgM in Bound and Unbound States. The average RMSD of the backbone particles for Fc and Fab domains of Matuzumab (in black) and Cetuximab (in red) IgM after least square fitting to the individual domain. The solid lines depict the mean values obtained from three independent 5 µs simulations, while the shaded regions denote the standard deviations. Results from *unbound* simulations are shown on the left, while *bound* simulation results are displayed on the right.

Supplementary Figure 2 Dominant Motions in Matuzumab and Cetuximab Systems Revealed by PCA. Porcupine plots of the first eigenvector are shown for three independent repeats of Matuzumab (top row) and Cetuximab (bottom row). Analysis was based on backbone atoms. Arrows indicate the direction of the principal motion (from thick to thin cones), and the color gradient (blue → green → red) represents increasing displacement, with red highlighting the regions undergoing the greatest conformational change.

Supplementary Figure 3 Fab-Fab Interaction May Induce Steric Hindrance That Can Prevent Simultaneous Fab–EGFR Binding. *Left:* Representative top-cluster structure (accounting for 65.7% of the total sampled configurations) derived from one of the Cetuximab simulations conducted without the antigen. All components are shown in surface representation, with the IgM subunit in cyan, the glycan residues in green, and the CDR and EGFR in magenta and yellow, respectively. *Right:* A zoom-in view of the first Fab domain of the IgM subunit bound to an EGFR monomer. Red circles highlight steric clashes between the exposed EGFR domain and the adjacent Fab region, thereby preventing the simultaneous binding of both Fab domains to the antigens.

Supplementary Figure 4 EGFR Alignment onto Cetuximab IgM Model. *Left:* Ten epidermal growth factor receptors (EGFRs) are aligned onto the Cetuximab IgM model, using the monomeric binding site detailed in the Methods section. The EGFRs are colored yellow and purple. Structural components of the IgM are differentiated by color: heavy chains in cyan, light chains in magenta, J chain in orange, and glycans in green. *Right:* This close-up view focuses on a single subunit of Cetuximab IgM with bound EGFRs. To enhance clarity, the visualization includes only two EGFR units and one IgM molecule. The IgM is displayed using a liquorice representation, with heavy and light chains in cyan and magenta, respectively, while the EGFRs are shown in surface representation and colored yellow and purple.

Supplementary Figure 5 Comparative Initial Setup of Domain III EGFR with IgM and IgG for Steered MD Simulations. The figure illustrates cartoon models of the initial setup of domain III EGFR, IgM, and IgG as used in steered MD simulations. Domain III of EGFR is colored purple, the antibody models are shown in blue, and the glycans are highlighted in green, with IgM on the left and IgG on the right.

Supplementary Figure 6 WHAM-Derived Histograms Across Umbrella Sampling Windows. Histograms generated from WHAM calculations represent the distribution of data across various US simulation windows. The x-axis represents the reaction coordinate, while the y-axis shows the observation count. Each histogram is color-coded to differentiate between the simulation windows.

## References

[1] Scott AM Wolchok JD Old LJ . Antibody therapy of cancer. Nat Rev Cancer. (2012) 12:278–87. doi: 10.1038/nrc3236, PMID: 22437872

[2] Weiner LM Surana R Wang S . Monoclonal antibodies: versatile platforms for cancer immunotherapy. Nat Rev Immunol. (2010) 10:317–27. doi: 10.1038/nri2744, PMID: 20414205 PMC3508064

[3] Carter PJ Lazar GA . Next generation antibody drugs: pursuit of the ‘high-hanging fruit,’. Nat Rev Drug Discov. (2018) 17:197–223. doi: 10.1038/nrd.2017.227, PMID: 29192287

[4] Kdimati S Mullins CS Linnebacher M . Cancer-cell-derived igG and its potential role in tumor development. Int J Mol Sci. (2021) 22:11597. doi: 10.3390/ijms222111597, PMID: 34769026 PMC8583861

[5] Callegari I Schneider M Berloffa G Mühlethaler T Holdermann S Galli E . Potent neutralization by monoclonal human IgM against SARS-CoV-2 is impaired by class switch. EMBO Rep. (2022) 23:2022. doi: 10.15252/embr.202153956, PMID: 35548920 PMC9253785

[6] Devito C Ellegård R Falkeborn T Svensson L Ohlin M Larsson M . Human IgM monoclonal antibodies block HIV-transmission to immune cells in cervico-vaginal tissues and across polarized epithelial cells in *vitro*. Sci Rep. (2018) 8:10180. doi: 10.1038/s41598-018-28242-y, PMID: 29977063 PMC6033918

[7] Rosenes Z Mulhern TD Hatters DM Ilag LL Power BE Hosking C . The anti-cancer igM monoclonal antibody PAT-SM6 binds with high avidity to the unfolded protein response regulator GRP78. PloS One. (2012) 7:e44927. doi: 10.1371/journal.pone.0044927, PMID: 23028685 PMC3446985

[8] Labrijn AF Janmaat ML Reichert JM Parren PWHI . Bispecific antibodies: a mechanistic review of the pipeline. Nat Rev Drug Discov. (2019) 18:585–608. doi: 10.1038/s41573-019-0028-1, PMID: 31175342

[9] Wu Y Yi M Zhu S Wang H Wu K . Recent advances and challenges of bispecific antibodies in solid tumors. Exp Hematol Oncol. (2021) 10:56. doi: 10.1186/s40164-021-00250-1, PMID: 34922633 PMC8684149

[10] Marei HE Hasan A Pozzoli G Cenciarelli C . Cancer immunotherapy with immune checkpoint inhibitors (ICIs): potential, mechanisms of resistance, and strategies for reinvigorating T cell responsiveness when resistance is acquired. Cancer Cell Int. (2023) 23:64. doi: 10.1186/s12935-023-02902-0, PMID: 37038154 PMC10088229

[11] Gonzalez-Quintela A Alende R Gude F Campos J Rey J Meijide LM . Serum levels of immunoglobulins (IgG, IgA, IgM) in a general adult population and their relationship with alcohol consumption, smoking and common metabolic abnormalities. Clin Exp Immunol. (2008) 151:42–50. doi: 10.1111/j.1365-2249.2007.03545.x, PMID: 18005364 PMC2276914

[12] Wang H Coligan JE Morse HC . Emerging functions of natural igM and its fc receptor FCMR in immune homeostasis. Front Immunol. (2016) 7:99. doi: 10.3389/fimmu.2016.00099, PMID: 27014278 PMC4791374

[13] Davis AC Shulman MJ . IgM - molecular requirements for its assembly and function. Immunol Today. (1989) 10:118–28. doi: 10.1016/0167-5699(89)90244-2, PMID: 2665773

[14] Brewer JW Randall TD Parkhouse RME Corley RB . IgM hexamers? Immunol Today. (1994) 15:165–8. doi: 10.1016/0167-5699(94)90313-1, PMID: 8198707

[15] Polonelli L Pontón J Elguezabal N Moragues MD Casoli C Pilotti E . Antibody complementarity-determining regions (CDRs) can display differential antimicrobial, antiviral and antitumor activities. PloS One. (2008) 3. doi: 10.1371/journal.pone.0002371, PMID: 18545659 PMC2396520

[16] Czajkowsky DM Shao Z . The human IgM pentamer is a mushroom-shaped molecule with a flexural bias. Proc Natl Acad Sci. (2009) 106:14960–5. doi: 10.1073/pnas.0903805106, PMID: 19706439 PMC2736442

[17] Cattaneo A Neuberger MS . Polymeric immunoglobulin M is secreted by transfectants of non-lymphoid cells in the absence of immunoglobulin J chain. EMBO J. (1987) 6:2753–8. doi: 10.1002/j.1460-2075.1987.tb02569.x, PMID: 3119328 PMC553699

[18] Niles MJ Matsuuchi L Koshland ME . Polymer IgM assembly and secretion in lymphoid and nonlymphoid cell lines: evidence that J chain is required for pentamer IgM synthesis. Proc Natl Acad Sci. (1995) 92:2884–8. doi: 10.1073/pnas.92.7.2884, PMID: 7708742 PMC42323

[19] Jutten B Keulers TG Schaaf MBE Savelkouls K Theys J Span PN . EGFR overexpressing cells and tumors are dependent on autophagy for growth and survival. Radiotherapy Oncol. (2013) 108:479–83. doi: 10.1016/j.radonc.2013.06.033, PMID: 23891088

[20] Tan X Lambert F Rapraeger AC Anderson XXXR. A . Stress-induced EGFR trafficking: mechanisms, functions, and therapeutic implications. Trends Cell Biol. (2016) 26:352–66. doi: 10.1016/j.tcb.2015.12.006, PMID: 26827089 PMC5120732

[21] Sigismund S Avanzato D Lanzetti L . Emerging functions of the EGFR in cancer. Mol Oncol. (2018) 12:3–20. doi: 10.1002/1878-0261.12155, PMID: 29124875 PMC5748484

[22] Schmiedel J Blaukat A Li S Knöchel T Ferguson KM . Matuzumab binding to EGFR prevents the conformational rearrangement required for dimerization. Cancer Cell. (2008) 13:365–73. doi: 10.1016/j.ccr.2008.02.019, PMID: 18394559 PMC2725356

[23] Wong. S-F . Cetuximab: An epidermal growth factor receptor monoclonal antibody for the treatment of colorectal cancer. Clin Ther. (2005) 27:684–94. doi: 10.1016/j.clinthera.2005.06.003, PMID: 16117976

[24] Samsudin F Yeo JY Gan SKE Bond PJ . Not all therapeutic antibody isotypes are equal: The case of IgM: Versus IgG in Pertuzumab and Trastuzumab. Chem Sci. (2020) 11:2843–54. doi: 10.1039/c9sc04722k, PMID: 32206268 PMC7069520

[25] Li Y Wang G Li N Wang Y Zhu Q Chu H . Structural insights into immunoglobulin M. Science. (2020) 367:1014–7. doi: 10.1126/science.aaz5425, PMID: 32029689

[26] Jefferis. R . Glycosylation of recombinant antibody therapeutics. Biotechnol Progress. (2005) 21:11–6. doi: 10.1021/bp040016j., PMID: 15903235

[27] Giddens JP Lomino JV DiLillo DJ Ravetch JV Wang LX . Site-selective chemoenzymatic glycoengineering of Fab and Fc glycans of a therapeutic antibody. Proc Natl Acad Sci U.S.A. (2018) 115:12023–7. doi: 10.1073/pnas.1812833115, PMID: 30397147 PMC6255169

[28] Janin-Bussat MC Tonini L Huillet C Colas O Klinguer-Hamour C Corvaïa N . Cetuximab fab and fc N-glycan fast characterization using ideS digestion and liquid chromatography coupled to electrospray ionization mass spectrometry. Biotechnol Progress (2013) 988:11–16. doi: 10.1007/978-1-62703-327-5_7., PMID: 23475716

[29] Qian J Liu T Yang L Daus A Crowley R Zhou Q . Structural characterization of N-linked oligosaccharides on monoclonal antibody cetuximab by the combination of orthogonal matrix-assisted laser desorption/ionization hybrid quadrupole–quadrupole time-of-flight tandem mass spectrometry and sequential enzymatic digestion. Anal Biochem. (2007) 364:8–18. doi: 10.1016/j.ab.2007.01.023, PMID: 17362871

[30] Arnold JN Wormald MR Suter DM Radcliffe CM Harvey DJ Dwek RA . Human serum IgM glycosylation: Identification of glycoforms that can bind to Mannan-binding lectin. J Biol Chem. (2005) 280:29080–7. doi: 10.1074/jbc.M504528200, PMID: 15955802

[31] Chen Q Menon R Calder LJ Tolar Rosenthal PB . Cryomicroscopy reveals the structural basis for a flexible hinge motion in the immunoglobulin M pentamer. Nat Commun. (2022) 13. doi: 10.1038/s41467-022-34090-2, PMID: 36274064 PMC9588798

[32] Akhouri RR Goel S Furusho H Skoglund U Wahlgren M . Architecture of human igM in complex with P. falciparum erythrocyte membrane protein 1. Cell Rep. (2016) 14:723–36. doi: 10.1016/j.celrep.2015.12.067, PMID: 26776517

[33] Lee HS Qi Y Im W . Effects of N-glycosylation on protein conformation and dynamics: Protein Data Bank analysis and molecular dynamics simulation study. Sci Rep. (2015) 5. doi: 10.1038/srep08926, PMID: 25748215 PMC4352867

[34] Sarkar A Wintrode PL . Effects of glycosylation on the stability and flexibility of a metastable protein: The human serpin α1-antitrypsin. Int J Mass Spectrom. (2011) 302:69–75. doi: 10.1016/j.ijms.2010.08.003, PMID: 21765645 PMC3134971

[35] Zheng K Bantog C Bayer R . The impact of glycosylation on monoclonal antibody conformation and stability. MAbs. (2011) 3. doi: 10.4161/mabs.3.6.17922, PMID: 22123061 PMC3242843

[36] Wolf B Piksa M Beley I Patoux A Besson T Cordier V . Therapeutic antibody glycosylation impacts antigen recognition and immunogenicity. Immunology. (2022) 166:380–407. doi: 10.1111/imm.13481, PMID: 35416297

[37] Liu. L . Antibody glycosylation and its impact on the pharmacokinetics and pharmacodynamics of monoclonal antibodies and fc-fusion proteins. J Pharm Sci. (2015) 104:1866–84. doi: 10.1002/jps.24444, PMID: 25872915

[38] Kumar N Arthur CP Ciferri C Matsumoto ML . Structure of the human secretory immunoglobulin M core. Structure. (2021) 29:564–571.e3. doi: 10.1016/j.str.2021.01.002, PMID: 33513362

[39] Chen Q Menon RP Masino) L . Tolar and P. B. Rosenthal., Structural basis for Fc receptor recognition of immunoglobulin M. Nat Struct Mol Biol. (2023) 30:1033–9. doi: 10.1038/s41594-023-00985-x, PMID: 37095205 PMC7614769

[40] Zhao J Nussinov R Ma B . Antigen binding allosterically promotes Fc receptor recognition. MAbs. (2019) 11:58–74. doi: 10.1080/19420862.2018.1522178, PMID: 30212263 PMC6343797

[41] Orlandi C Deredge D Ray K Gohain N Tolbert W DeVico AL . Antigen-induced allosteric changes in a human igG1 fc increase low-affinity fcγ Receptor binding. Structure. (2020) 28:516–527.e5. doi: 10.1016/j.str.2020.03.001, PMID: 32209433 PMC7288244

[42] Lua WH Tran-To Su C Yeo JY Poh JJ Ling WL Phua SX . Role of the IgE variable heavy chain in FcϵRIα and superantigen binding in allergy and immunotherapy. J Allergy Clin Immunol. (2019) 144:514–523.e5. doi: 10.1016/j.jaci.2019.03.028, PMID: 30995457

[43] Janda A Bowen A Greenspan NS Casadevall A . Ig constant region effects on variable region structure and function. Feb. 04, 2016. Front Media S.A. (2016) 22. doi: 10.3389/fmicb.2016.00022, PMID: 26870003 PMC4740385

[44] Su CTT Lua WH Ling WL Gan SKE . Allosteric effects between the antibody constant and variable regions: A study of IgA Fc mutations on antigen binding. Antibodies. (2018) 7. doi: 10.3390/antib7020020, PMID: 31544872 PMC6698812

[45] Hale G Bright S Chumbley G Hoang T Metcalf D Munro AJ . Removal of T cells from bone marrow for transplantation: a monoclonal antilymphocyte antibody that fixes human complement. Blood. (1983) 62:873–82., PMID: 6349718

[46] Gong S Tomusange K Kulkarni V Adeniji OS Lakhashe SK Hariraju D . Anti-HIV IgM protects against mucosal SHIV transmission. AIDS. (2018) 32:F5–F13. doi: 10.1097/QAD.0000000000001857, PMID: 29762161 PMC6380498

[47] Irie RF Ollila DW O’Day S Morton DL . Phase I pilot clinical trial of human IgM monoclonal antibody to ganglioside GM3 in patients with metastatic melanoma. Cancer Immunology Immunotherapy. (2004) 53:110–7. doi: 10.1007/s00262-003-0436-1, PMID: 14564483 PMC11034296

[48] Brändlein S Rauschert N Rasche L Dreykluft A Hensel F Conzelmann E . The human IgM antibody SAM-6 induces tumor-specific apoptosis with oxidized low-density lipoprotein. Mol Cancer Ther. (2007) 6:326–33. doi: 10.1158/1535-7163.MCT-06-0399, PMID: 17237291

[49] Liedtke M Twist CJ Medeiros BC Gotlib JR Berube C Bieber MM . Phase I trial of a novel human monoclonal antibody mab216 in patients with relapsed or refractory B-cell acute lymphoblastic leukemia. Haematologica. (2012) 97:30–7. doi: 10.3324/haematol.2011.045997, PMID: 21993685 PMC3248928

[50] Rasche L Duell J Castro IC Dubljevic V Chatterjee M Knop S . GRP78-directed immunotherapy in relapsed or refractory multiple myeloma - results from a phase 1 trial with the monoclonal immunoglobulin M antibody PAT-SM6. Haematologica. (2015) 100:377–384, 2015. doi: 10.3324/haematol.2014.117945, PMID: 25637055 PMC4349277

[51] Rasche L Duell J Morgner C Chatterjee M Hensel F Rosenwald A . The natural human igM antibody PAT-SM6 induces apoptosis in primary human multiple myeloma cells by targeting heat shock protein GRP78. PloS One. (2013) 8. doi: 10.1371/journal.pone.0063414, PMID: 23667612 PMC3646784

[52] Vollmers HP Brändlein S . Nature’s best weapons to fight cancer. Revival of human monoclonal IgM antibodies. Hum Antibodies. (2003) 11:131–42. doi: 10.3233/HAB-2002-11403, PMID: 12775893

[53] Periole X Knepp AM Sakmar TP Marrink SJ Huber T . Structural determinants of the supramolecular organization of G protein-coupled receptors in bilayers. J Am Chem Soc. (2012) 134:10959–65. doi: 10.1021/ja303286e, PMID: 22679925 PMC3406292

[54] Koldsø H Sansom MSP . Organization and dynamics of receptor proteins in a plasma membrane. J Am Chem Soc. (2015) 137:14694–704. doi: 10.1021/jacs.5b08048, PMID: 26517394 PMC5591644

[55] Xing PX Hu XF Pietersz GA Hosick HL McKenzie IF . Cripto: a novel target for antibody-based cancer immunotherapy. Cancer Res. (2004) 64:4018–23. doi: 10.1158/0008-5472.CAN-03-3888, PMID: 15173016

[56] Li S Schmitz KR Jeffrey D Wiltzius JJW . Kussie and K. M. Ferguson., Structural basis for inhibition of the epidermal growth factor receptor by cetuximab. Cancer Cell. (2005) 7:301–11. doi: 10.1016/j.ccr.2005.03.003, PMID: 15837620

[57] Müller R Gräwert MA Kern T Madl T Peschek J Sattler M . High-resolution structures of the IgM Fc domains reveal principles of its hexamer formation. Proc Natl Acad Sci. (2013) 110:10183–8. doi: 10.1073/pnas.1300547110, PMID: 23733956 PMC3690842

[58] Lobner E Humm AS Göritzer K Mlynek G Puchinger MG Hasenhindl C . Fcab-HER2 interaction: a ménage à Trois. Lessons from X-ray and solution studies. Structure. (2017) 25:878–889.e5. doi: 10.1016/j.str.2017.04.014, PMID: 28528777

[59] Harris LJ Larson SB Hasel KW McPherson A . Refined structure of an intact igG2a monoclonal antibody. Biochemistry. (1997) 36:1581–97. doi: 10.1021/bi962514+, PMID: 9048542

[60] Schrödinger LLC . The pyMOL molecular graphics system, version~1.8. (2015).

[61] Šali A Blundell TL . Comparative protein modelling by satisfaction of spatial restraints. J Mol Biol. (1993) 234:779–815. doi: 10.1006/jmbi.1993.1626, PMID: 8254673

[62] Huang Y Ognjenovic J Karandur D Miller K Merk A Subramaniam S . A molecular mechanism for the generation of ligand-dependent differential outputs by the epidermal growth factor receptor. Elife. (2021) 10. doi: 10.7554/eLife.73218, PMID: 34846302 PMC8716103

[63] Abraham MJ Murtola T Schulz R Páll S Smith JC Hess B . Gromacs: High performance molecular simulations through multi-level parallelism from laptops to supercomputers. SoftwareX. (2015) 1:1–2. doi: 10.1016/j.softx.2015.06.001

[64] Periole X Cavalli M Marrink S-J Ceruso MA . Combining an elastic network with a coarse-grained molecular force field: structure, dynamics, and intermolecular recognition. J Chem Theory Comput. (2009) 5:2531–43. doi: 10.1021/ct9002114, PMID: 26616630

[65] Monticelli L Kandasamy SK Periole X Larson RG Tieleman DP Marrink S-J . The MARTINI coarse-grained force field: extension to proteins. J Chem Theory Comput. (2008) 4:819–34. doi: 10.1021/ct700324x, PMID: 26621095

[66] Shivgan AT Marzinek JK Huber RG Krah A Henchman RH Matsudaira P . Extending the martini coarse-grained force field to *N* -glycans. J Chem Inf Model. (2020) 60:3864–83. doi: 10.1021/acs.jcim.0c00495, PMID: 32702979

[67] Arnold JN Wormald MR Suter DM Radcliffe CM Harvey DJ Dwek RA . Human serum IgM glycosylation: Identification of glycoforms that can bind to Mannan-binding lectin. J Biol Chem. (2005) 280:29080–7. doi: 10.1074/jbc.M504528200, PMID: 15955802

[68] Bussi G Donadio D Parrinello M . Canonical sampling through velocity rescaling. J Chem Phys. (2007) 126. doi: 10.1063/1.2408420, PMID: 17212484

[69] Berendsen HJC Postma JPM van Gunsteren WF DiNola A Haak JR . Molecular dynamics with coupling to an external bath. J Chem Phys. (1984) 81:3684–90. doi: 10.1063/1.448118

[70] Parrinello M Rahman A . Polymorphic transitions in single crystals: A new molecular dynamics method. J Appl Phys. (1981) 52:7182–90. doi: 10.1063/1.328693

[71] Humphrey W Dalke A Schulten K . VMD: Visual molecular dynamics. J Mol Graph. (1996) 14:33–8. doi: 10.1016/0263-7855(96)00018-5, PMID: 8744570

[72] Kumar S Rosenberg JM Bouzida D Swendsen RH Kollman PA . THE weighted histogram analysis method for free-energy calculations on biomolecules. I. The method. J Comput Chem. (1992) 13:1011–21. doi: 10.1002/jcc.540130812

[73] Hub JS de Groot BL van der Spoel D . g_wham—A free weighted histogram analysis implementation including robust error and autocorrelation estimates. J Chem Theory Comput. (2010) 6:3713–20. doi: 10.1021/ct100494z

