# Supplementary material for: Targeting the epidermal growth factor receptor using IgM antibodies: toward next generation cancer immunotherapy
Source: Front Immunol. 2026 Jan 9;16:1733907. doi: 10.3389/fimmu.2025.1733907 (PMC12827627; doi:10.3389/fimmu.2025.1733907)
Supplement: Supplementary Table 1 — List of all production simulations [file DataSheet1.pdf]

Supplementary Materials

Supplementary Table 1: List of all production simulations

Antibody		No. of Antigens	Equilibrium simulations		Umbrella Sampling (US)	
			Number of simulations	Simulation time (μs)	Number of windows	Simulation time (μs)/window
IgG	Unbound Cetuximab		3	5		
	Bound Cetuximab	10	3	5		
	US Cetuximab	1			80	0.5
	Unbound Matuzumab		3	5		
	Bound Matuzumab	10	3	5		
	US Matuzumab	1			80	0.5
IgM	Unbound Cetuximab		3	5		
	Bound Cetuximab	10	3	5		
	US Cetuximab	1			80	0.5
	Unbound Matuzumab		3	5		
	Bound Matuzumab	10	3	5		
	US Matuzumab	1			80	0.5

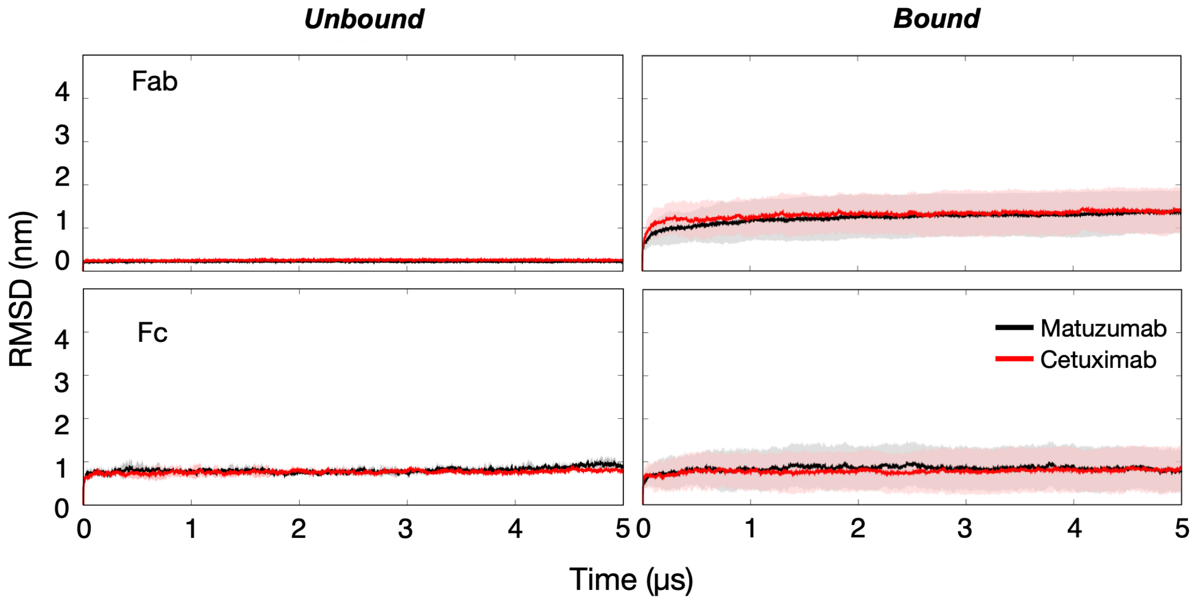

Supplementary Figure 1: RMSD Profiles for Matuzumab and Cetuximab IgM in Bound and Unbound States. The average RMSD of the backbone particles for Fc and Fab domains of Matuzumab (in black) and Cetuximab (in red) IgM after least square fitting to the individual domain. The solid lines depict the mean values obtained from three independent 5 μs simulations, while the shaded regions denote the standard deviations. Results from *unbound* simulations are shown on the left, while *bound* simulation results are displayed on the right.

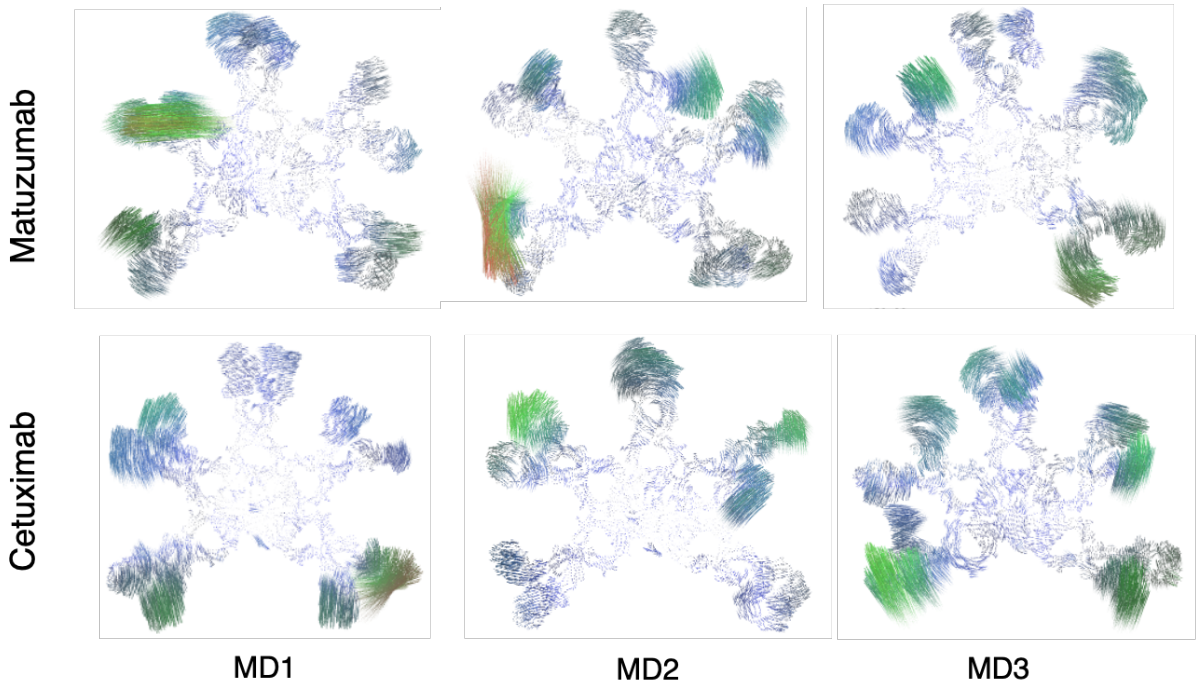

Supplementary Figure 2: **Dominant Motions in Matuzumab and Cetuximab Systems Revealed by PCA.** Porcupine plots of the first eigenvector are shown for three independent repeats of Matuzumab (top row) and Cetuximab (bottom row). Analysis was based on backbone atoms. Arrows indicate the direction of the principal motion (from thick to thin cones), and the colour gradient (blue → green → red) represents increasing displacement, with red highlighting the regions undergoing the greatest conformational change.

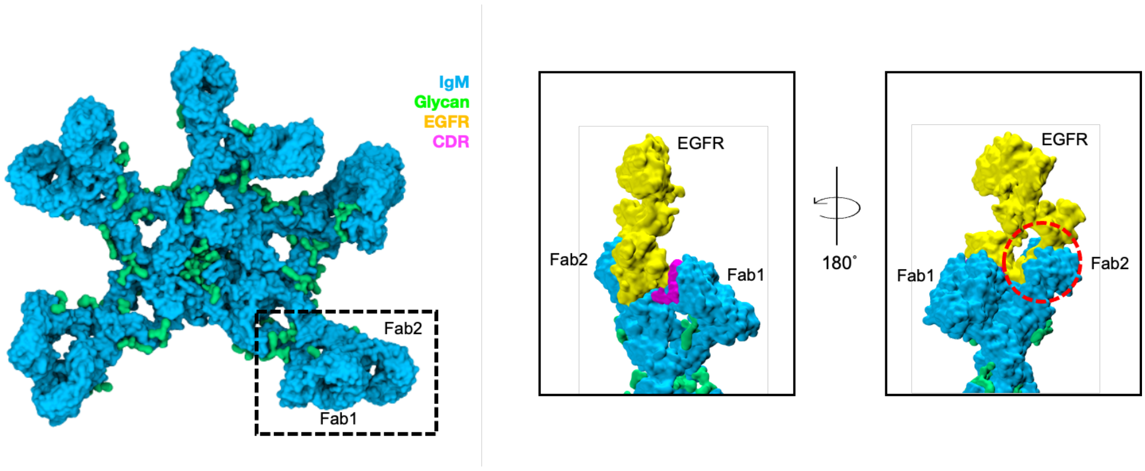

Supplementary Figure 3: **Fab-Fab Interaction May Induce Steric Hindrance That Can Prevent Simultaneous Fab–EGFR Binding.** ***Left:*** Representative top-cluster structure (accounting for 65.7% of the total sampled configurations) derived from one of the Cetuximab simulations conducted without the antigen. All components are shown in surface representation, with the IgM subunit in cyan, the glycan residues in green, and the CDR and EGFR in magenta and yellow, respectively. ***Right:*** A zoom-in view of the first Fab domain of the IgM subunit bound to an EGFR monomer. Red circles highlight steric clashes between the exposed EGFR domain and the adjacent Fab region, thereby preventing the simultaneous binding of both Fab domains to the antigens.

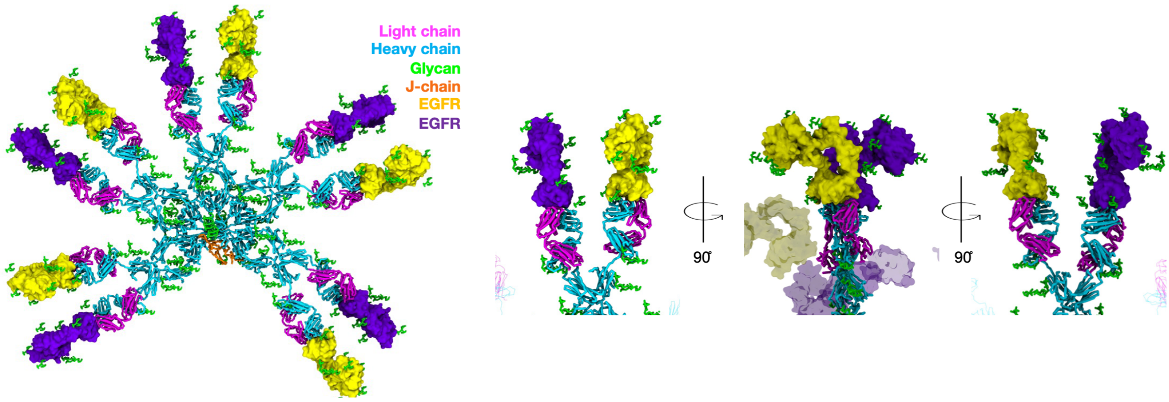

Supplementary Figure 4: EGFR Alignment onto Cetuximab IgM Model. ***Left:*** Ten epidermal growth factor receptors (EGFRs) are aligned onto the Cetuximab IgM model, using the monomeric binding site detailed in the Methods section. The EGFRs are coloured yellow and purple. Structural components of the IgM are differentiated by colour: heavy chains in cyan, light chains in magenta, J chain in orange, and glycans in green. ***Right:*** This close-up view focuses on a single subunit of Cetuximab IgM with bound EGFRs. To enhance clarity, the visualisation includes only two EGFR units and one IgM molecule. The IgM is displayed using a liquorice representation, with heavy and light chains in cyan and magenta, respectively, while the EGFRs are shown in surface representation and coloured yellow and purple.

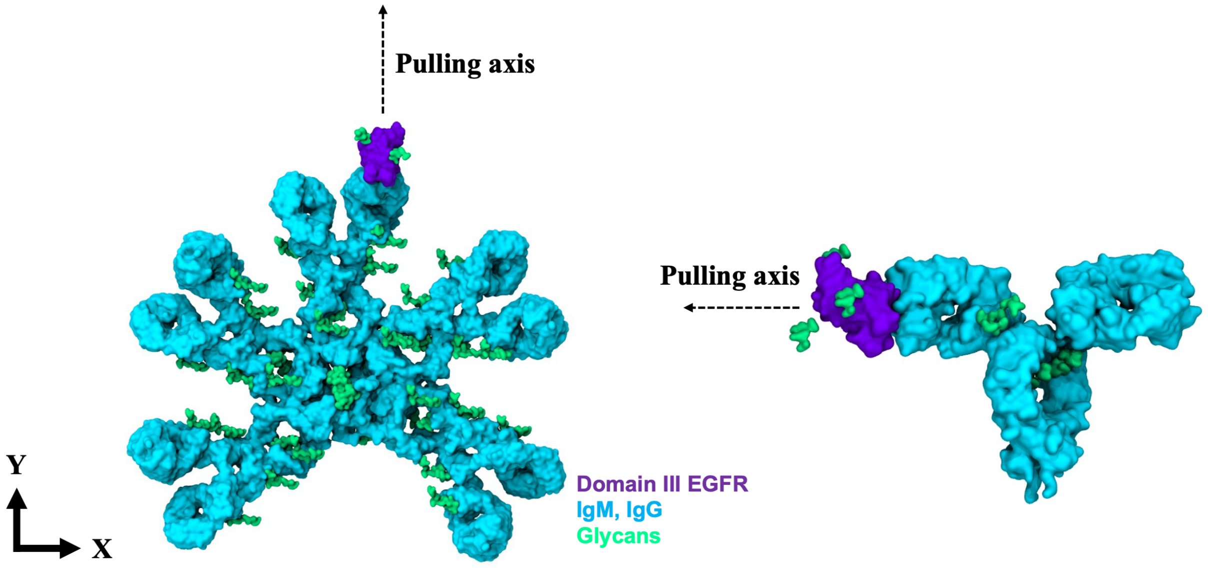

Supplementary Figure 5: Comparative Initial Setup of Domain III EGFR with IgM and IgG for Steered MD Simulations. The figure illustrates cartoon models of the initial setup of domain III EGFR, IgM, and IgG as used in steered MD simulations. Domain III of EGFR is coloured purple, the antibody models are shown in blue, and the glycans are highlighted in green, with IgM on the left and IgG on the right.

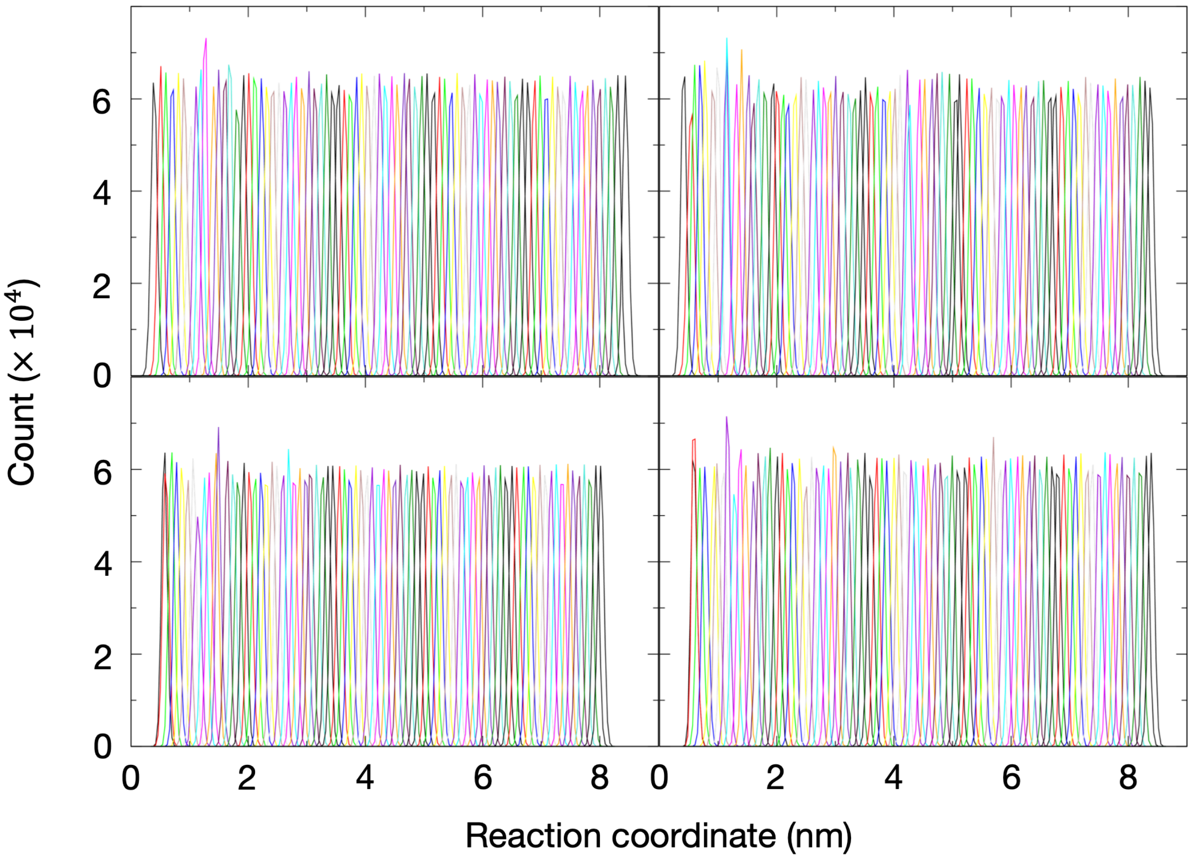

Supplementary Figure 6: WHAM-Derived Histograms Across Umbrella Sampling Windows. Histograms generated from WHAM calculations represent the distribution of data across various US simulation windows. The x-axis represents the reaction coordinate, while the y-axis shows the observation count. Each histogram is color-coded to differentiate between the simulation windows.
